# Early detection in oral cancer: Are we prepared for artificial intelligence-driven precision medicine?

**DOI:** 10.1097/MS9.0000000000004397

**Published:** 2025-11-24

**Authors:** Nikil Kumar, Sinnha Kumari, Sadia Sultana, Muhammad Saad Khan, Tirath Patel, Nikhilesh Anand

**Affiliations:** a Department of Medicine, Liaquat University of Medical and Health Sciences (LUMHS), Jamshoro, Pakistan; b Department of Medicine, Azad Jammu And Kashmir Medical College, Muzaffarabad, Azad Kashmir, Pakistan; c Department of Medicine, Jinnah Sindh Medical University, Karachi, Pakistan; d Department of Surgery, Trinity Medical Sciences University School of Medicine, Saint, Vincent and the Grenadines; e Department of Medical Education, University of Texas Rio Grande Valley, Edinburg, TX, USA

**Keywords:** artificial intelligence, oral cancer care, personalized treatment

## Abstract

Oral cancer, particularly oral squamous cell carcinoma, remains a serious health concern, with a poor prognosis and a late diagnosis. Leukoplakia, erythroplakia, lichen planus, and submucous fibrosis are examples of oral potentially malignant disorders that must be detected early but are not always so by traditional, laborious, and subjective diagnostic techniques. In oral cancer, artificial intelligence (AI) and precision medicine are becoming game-changing technologies that enhance individualized care, treatment planning, and diagnostic precision. Complex imaging and histopathology data may be analyzed using machine learning and deep learning algorithms, particularly convolutional neural networks, which can identify patterns that are invisible to the human eye. AI systems based on smartphones have demonstrated expert-level accuracy in identifying oral lesions in recent experiments. Through the discovery of biomarkers and the integration of several omics, AI-driven precision medicine also makes customized treatments possible. Nonetheless, issues with patient privacy, data bias, and the opaque “black box” nature of AI systems persist. The future of proactive, individualized oral cancer care will be shaped by the development of Explainable AI and robust ethical frameworks, both of which are necessary to ensure transparency, trust, and equitable integration.

*Dear Editor,*

Oral cancer remains one of the most challenging malignancies, with poor prognosis and substantial morbidity despite advances in therapy^[1]^. The most common form, oral squamous cell carcinoma (OSCC), is often preceded by oral potentially malignant disorders (OPMDs), such as leukoplakia, erythroplakia, lichen planus, and submucous fibrosis^[2]^. Detecting these disorders early is critical, yet conventional diagnostic tools such as oral examination, biopsy, and histopathology are slow, subjective, and prone to fluctuation. The ongoing reliance on these procedures contributes to late diagnosis, when therapeutic options are restricted and survival is compromised. This manuscript follows the TITAN guidelines to explore the use of Artificial Intelligence (AI) and precision medicine for the diagnostic, therapeutic, and early detection of oral squamous cell carcinoma (OSCC)^[3]^.

AI and precision medicine represent a revolutionary prospect in oral oncology. AI-powered technologies are improving the precision, repeatability, and treatment planning of diagnostics^[4]^. Machine learning and deep learning algorithms can analyze enormous complex datasets spanning fluorescence to hyperspectral imaging, revealing patterns beyond human detection^[2]^. Convolutional neural networks, in particular, hold promise for automating histopathology and integrating multimodal imaging, but their reliance on large, homogeneous datasets raises questions about bias and generalizability^[5]^.

Notably, a recent trial assessing a deep learning system installed on a smartphone for the detection of oral cancer and possibly malignant conditions demonstrates the usefulness of AI. The system was evaluated in three settings: independently, aiding dentists, and supporting non-certified health care, and achieved diagnosis accuracy comparable to that of an expert^[6]^. In actuality, these innovations enable more accurate tumor delineation, earlier detection, and treatment plans tailored to each patient’s molecular and genetic characteristics^[4]^.

Potentialities extend beyond diagnosis. Precision medicine, aided by artificial intelligence, is poised to transform the treatment of oral cancer. Algorithm-driven analytics and predictive biomarkers can direct immunotherapy and tailored treatments, boosting effectiveness and lowering toxicity. While biomarker-driven precision medicine uses single molecular fingerprints to guide targeted therapeutics, systems biology recognizes the complexity of interconnected networks across genomes, proteomics, and clinical characteristics. AI has the unique opportunity to improve both by expediting biomarker development and by integrating multi-omics to illuminate the broader tumor ecology via deep learning. Matching patients to the most effective strategy based on their individual tumor biology has the potential to transform oral oncology from a reactive to a proactive field^[4]^. For instance, integrating genomic and proteomic data through AI has facilitated the identification of salivary biomarkers linked to early OSCC progression, offering a non-invasive diagnostic adjunct.

Although there is significant promise for AI in oncology, the “black box” nature of many AI systems remains a significant ethical concern. It is crucial to protect patient data privacy, reduce algorithmic bias by using a variety of datasets, improve model explainability, and build up explicit legal frameworks. The creation of Explainable AI (XAI) is essential to guarantee transparency and confidence in AI-driven decision-making^[7]^. XAI is crucial for making critical judgments, especially in clinical settings, where the wrong choice can have catastrophic consequences, including loss of human life^[8]^.

The future of AI in oral oncology will depend not only on algorithmic sophistication but also on equitable access, regulatory oversight, and responsible integration into clinical workflows. Collaboration among clinicians, computer scientists, and policymakers is essential to translate these innovations from research to real-world impact. With proper governance and ethical safeguards, AI and precision medicine can shift oral cancer care from reactive management toward proactive personalized intervention, transforming outcomes and saving lives.

## Data Availability

No new dataset was generated during the study.

## Research registration unique identifying number (UIN)

Not required because the study is a letter to the editor.

## References

[1] García-Pola M Pons-Fuster E Suárez-Fernández C. Role of Artificial Intelligence in the early diagnosis of oral cancer. A scoping review. Cancers (Basel) 2021;13:4600. 34572831 10.3390/cancers13184600 PMC8467703

[2] Hegde S Ajila V Zhu W. Artificial intelligence in early diagnosis and prevention of oral cancer. Asia-Pac J Oncol Nurs 2022;9:100133. 36389623 10.1016/j.apjon.2022.100133 PMC9664349

[3] Agha RA Mathew G Rashid R. TITAN Group. Transparency in the reporting of Artificial INtelligence – the TITAN guideline. Prem J Sci 2025;10:100082.

[4] Kapoor DU Saini PK Sharma N. AI illuminates paths in oral cancer: transformative insights, diagnostic precision, and personalized strategies. EXCLI J 2024;23:1091–116. 39391057 10.17179/excli2024-7253 PMC11464865

[5] Al-Rawi N Sultan A Rajai B. The effectiveness of Artificial Intelligence in detection of oral cancer. Int Dent J 2022;72:436–47. 35581039 10.1016/j.identj.2022.03.001 PMC9381387

[6] National Taiwan University Hospital. Application and validation of a smartphone-based deep learning system for Oral Potentially Malignant Disorders (OPMD) and oral cancer screening. clinicaltrials.gov. Report No.: NCT06862414. Accessed 19 August 2025. https://clinicaltrials.gov/study/NCT06862414

[7] Bongurala AR Save D Virmani A. Progressive role of artificial intelligence in treatment decision-making in the field of medical oncology. Front Med 2025;12:1533910. 10.3389/fmed.2025.1533910 PMC11865077 40018354

[8] Muhammad D Bendechache M. Unveiling the black box: a systematic review of explainable Artificial Intelligence in medical image analysis. Comput Struct Biotechnol J 2024;24:542–60. 39252818 10.1016/j.csbj.2024.08.005 PMC11382209

